# A Reference Hand-book of the Medical Sciences

**Published:** 1886-05

**Authors:** 


A Reference Handbook of the Medical Sciences, embracing the entire range of scientific and practical medicine and allied sciences by various writers. Illustrated by chromo-lithographs and fine wood engravings. Edited by Albert H. Buck, New York city. Vol. II. New York: William Wood & Company, 56 and 58 Lafayette Place. 1886.

Having already given full details of this comprehensive undertaking to furnish practical and scientific information upon the various topics connected with medicine, as evinced in the first volume of this work, we have only now to note the further progress of the enterprise in this second volume.

Among the many interesting articles, we are pleased to notice valuable contributions from members of the profession in our State—Papers on Dengue Fever and Enemata, by Dr. Eugene Foster, of Augusta.

The paper on Dengue Fever, by Dr. Foster, is a valuable contribution to the literature of the subject. He has gone fully into the subject and has left nothing unsaid. Of the treatment of this painful disease Dr. Foster says quinine is claimed to be a prophylactic when taken in doses of ten or fifteen grains daily. He unqualifiedly denies this. He tested it thoroughly in his own person and it proved of no benefit. He says that judicious symptomatic treatment can do much to mitigate the high fever and extreme suffering which so debilitates the patient.

Of the various drugs administered for antipyretic effect, he says he knows of none equal to quinine in thirty to forty-five grain doses within from two to four hours to adults; within six hours after taking full doses of quinine diminution of the fever will be observed.

The paper of Dr. Foster, on enemata, while not a lengthy one, is exhaustive. He clearly shows that benefit is derived from the proper administration of food by the rectum, and lays down a number of rules to be observed in administering the enemata. He says the rectum should be washed out once daily, and that the food should always be of the same temperature as the body. From four to six ounces should be used at one time if placed in the rectum, eight to twelve if in the colon. He especially urges that patients should be kept perfectly quiet in the recumbent position for at least thirty minutes after receiving the enema.

Twenty-two pages are occupied with Cholecystectomy, Cholecystotomy and Duodeno-Cholecystostomy, by Dr. J. McF. Gaston, of Atlanta.

These subjects cover a wide field of thought, and have been handled in a masterly manner by Dr. Gaston. The paper on cholecystectomy concludes as follows : Although twenty-five of the thirty-three completed operations of cholecystotomy have been done by English and American surgeons, it is a notable fact that cholecystectomy has not thus far been undertaken in a single case in Great Britain or the United States; but it only requires to be properly understood to be appreciated by surgeons.

The paper on cholecystotomy is very full and complete. It concludes with a tabulated statement that gives a complete record of authenticated cases in which operations have been undertaken. From this statement we note that the mortality of unfinished operations reaches forty per cent., there being two deaths out of five cases, while the fatality in completed operations of cholecystotomy is 27-27 per cent., nine having died out of thirty-three cases; and cholecystectomy gives but 12-50 per cent. of mortality, one death in eight cases of extirpation of the gall-bladder.

Duodeno–cholecystostomy occupies seven pages of the book. This operation was first suggested and described by Dr. Gaston in THE JOURNAL for September, 1884. The paper contains facts relating to numerous experiments made on dogs, and contains many original drawings by the author, demonstrating the feasibility of the operation as suggested.

At the close of the paper the author sums up the inferences to be drawn from these experiments, the chief of which is that the walls of the gall-bladder under such conditions contract and partake of the nature of a conduit, so that the biliary secretion is urged forward and prevents the ingress of the alimentary mass from the duodenal canal. The constant flow of bile through the orifice must preserve it open, as in a cutaneous fistula, and maintain a permanent communication between the gall-bladder and duodenum.

Other valuable disquisitions upon various topics by representative men in the several departments render this volume highly useful as a book of reference, and we commend it to the careful study of all who are interested in the progress of medical sciences.

We observe among the contributions the following live questions of the day are thoroughly considered : Acute and chronic nasal catarrh, cerebral functions, cerebro-spinal meningitis, chancre, cleft palate, club-foot, coca, cremation, diabetes mellitus, diphtheria, dislocations, ear troubles, epilepsy, evolution, extra-uterine pregnancy, expert testimony, affections of the eyes, with other practical subjects which our space does not permit us to notice.

This volume is executed in a style of excellence that sustains the well-earned celebrity of the publishers in book-making, and affords ample evidence of success in their present laudable effort to supply the medical profession with a most valuable encyclopædia of practical and scientific literature.

